# Enteric fever due to Salmonella Paratyphi A in Greece: a case report

**DOI:** 10.1186/1757-1626-1-403

**Published:** 2008-12-16

**Authors:** Athanasios Chalkias, Dimitrios Anastasopoulos, Stavros Tsiaglis

**Affiliations:** 1 Department of General Medicine, Tzaneio Hospital, Piraeus, Greece; 2 Department of Pathology, 1^st ^Pathological Clinic, Tzaneio Hospital, Piraeus, Greece

## Abstract

**Background:**

Enteric fever is a major global public health problem and growing international travel leads to an increase in the risk of contracting infectious diseases that are endemic in the country of destination.

**Case presentation:**

A 30-year-old Pakistani male was admitted feeling generally unwell with fever, vertigo, vomiting and headache. Admission blood cultures grew Salmonella ser. Paratyphi A. The patient was successfully treated with intravenous Amoxicillin/Clavulanic Acid and Ciprofloxacin.

**Conclusion:**

This case report emphasizes the potent spread of Salmonellae bacilli in a developed country.

## Background

Typhoid fever is the most serious form of enteric fever, with humans being the sole reservoir of the bacteria. Based on a recent survey, the global number of typhoid cases in 2000 exceeded 21,000,000, with more than 200,000 deaths [1]. Enteric fever, that is typhoid and paratyphoid fevers, is the common name for infections caused by Salmonella enterica serotypes typhi and paratyphi. Of the three types of S. paratyphi (A, B, and C), B is the most common.

Salmonellae are motile, gram-negative, non-spore-forming bacilli and can be differentiated into more than 2000 serotypes (serovars) by their somatic (O) antigens. There are six serogroups: A, B, C1, C2, D, and E. Although any Salmonella serotype can produce any of the Salmonella syndromes each serotype tends to be associated with certain syndromes much more frequently than others. S. typhi and S. paratyphi are most likely to cause enteric fever, as well as the chronic carrier state. There are three species of Salmonellae that cause paratyphoid: Salmonella paratyphi A, S. paratyphi B (or S. schotmulleri) and S. paratyphi C (S. hirschfeldii) [2]. As a result of modern sewage and water treatment facilities, these diseases have become rare in developed countries but remain a problem in countries without adequate sanitation and a safe water supply. Although enteric fever is a major global public health problem, data on the relative risk of contracting travel – associated enteric fever in various regions of the world are scarce. Growing international travel leads to an increase in the risk of contracting infectious diseases that are endemic in the country of destination [3].

Salmonella infection in humans usually occurs from pets, other humans and from ingesting contaminated water or animal food products, most often eggs, poultry, and meat [2,4,5]. Any food can become contaminated by feces. After the ingestion of organisms, the likelihood of infection developing, as well as the severity of infection, is related to the dose and virulence of the Salmonella strain and the status of host defense mechanisms. Although any Salmonella serotype can produce the syndrome of bacteremia, S. choleraesuis and S. dublin are most likely to cause this syndrome.

The asymptomatic intestinal carrier state may result from inapparent infection or may follow clinical disease. It is usually self-limited to several weeks to months, with the incidence of positive stool cultures rapidly decreasing over time. The syndrome of enteric fever is characterized by prolonged sustained fever, relative bradycardia, hepatosplenomegaly, rose spots, and leucopenia and neutropenia [3]. After an incubation period of 5 to 21 days (generally 7 to 14 days), fever and malaise develop, often associated with cough. A small proportion of patients may have diarrhea during the incubation period. The fever tends to rise in stepwise fashion over the first few days to a week and then becomes sustained, usually at 39.4 to 40°C (103 to 104°F) or higher. After 2 weeks of illness, the severe complications of intestinal hemorrhage or perforation may be observed. The illness usually resolves by the end of the fourth week in an untreated patient. Relapse may occur in untreated as well as treated patients, but the illness is milder than the original episode. Rarely, some of the following complications may occur: pancreatitis, cholecystitis, infective endocarditis, pneumonia, hepatic or splenic abscess, orchitis, or focal infection at virtually any site [6].

The differential diagnosis of enteric fever is very broad and depends in part on the area of the world in which the infection was acquired. The differential diagnosis of Salmonella bacteremia includes all acute infectious and noninfectious causes of fever, including bacteremia caused by other organisms.

The diagnosis is proved by isolation of the microorganism from blood or a site of localization. The diagnosis of enteric fever is best proved by isolation of the microorganism from blood, stool, or bone marrow. During the first week of illness, blood cultures are positive in about 90% of patients, but culture positivity decreases over the next 2 weeks to less than 50% during the third week of illness. Stool cultures are usually negative during the first week but by the third week are generally positive. Bone marrow cultures give the highest yield, with up to 95% being positive, and should be obtained in suspected cases with negative blood cultures.

The primary approach to treatment of Salmonella enterocolitis is fluid and electrolyte replacement, correction of acid-base disturbances, and in the setting of intestinal bleeding, blood transfusion. Drugs with antiperistaltic effects such as loperamide can relieve cramps but can prolong the diarrhea. The fluoroquinolones can be used against Salmonellae [3]. A third-generation cephalosporin is an alternative. Other agents, such as amoxicillin, chloramphenicol, ampicillin, azithromycin and trimethoprim-sulfamethoxazole, have also been widely used in severely ill adults. However, many strains of Salmonella are now resistant to these agents which results in poor clinical outcomes [6-8]. In the presence of gross bloody diarrhea, antimicrobial therapy should be withheld until the possibility of E. coli O157:H7 infection has been eliminated because antibiotic therapy may increase the frequency of development of hemolytic-uremic syndrome. If perforation seems likely, laparotomy should be performed as soon as possible to repair the perforation. Steroid therapy has shown to be beneficial in some patients with severe enteric fever and coma, delirium, or shock. Steroids can mask the signs and symptoms of abdominal perforation and should not be continued for more than 48 hours. Salicylates should be avoided. Relapses of typhoid fever should be treated with the same antimicrobial regimen as the initial attack. In cases of sustained bacteremia, the possibility of endovascular infection should be investigated. For transient bacteremia or bacteremia without localization, therapy is continued for 7 to 14 days. With localization to bone, aneurysms, heart valves, and various other sites, antimicrobial therapy should be given for much longer periods (e.g. 6 weeks). Surgical drainage, removal of foreign bodies, or resection of an aneurysm is often necessary to cure localized infection.

Mortality from Salmonella is not uncommon and is most likely to occur in the very young, the very old, and the immunocompromised. The mortality rate remains as high as 30 to 50% in some areas of developing countries. With perforation, mortality rates of 10 to 30% have been reported. Up to 3% of patients recovering from S. typhi infection become chronic fecal carriers.

## Case presentation

A 30-year-old Pakistani male was admitted feeling generally unwell with fever, vertigo, vomiting and headache. The onset of fever was 3 days after his return from Pakistan. His medical history was unremarkable.

His temperature was 41.5°C, blood pressure 135/75 mm Hg, heart rate 109 beats min^-1^. Physical examination was unremarkable.

Analysis of blood showed haemoglobin 13.3 g/DL, white cell count 3.57 × 10^9 ^L^-1^, neutrophils 5.3 × 10^9 ^L^-1^, Platelets 30 × 10^9 ^L^-1^, Na^+ ^133 meq/L, SGOT 57 U/L, SGPT 57 U/L, LDH 305 U/L, CPK 252 U/L, C – reactive protein 75,4 mg/L. Urinalysis and urine culture, chest radiogram, viral and immunological tests, fecal tests, abdominal ultrasound, thyroid function, the Mantoux test, blood gases, tumor markers were normal and electrocardiogram revealed a sinus tachycardia. Treatment was initiated with intravenous fluid replacement and three sets of blood cultures were taken from different veins. On day 2 of admission the organism was identified as Salmonella ser. Paratyphi A, and was resistant to Cefazolin, Cefuroxime and Aminoglycosides. Antimicrobial therapy was initiated with Amoxicillin/Clavulanic Acid 1.2 g 8 – hourly (based on antibiogram) because the patient could not tolerate oral therapy.

The patient's condition failed to improve and he continued to spike temperatures of up to 39.5°C. On day 5 of his admission, his condition was stable and the blood analysis showed SGOT 399 U/L, SGPT 438 U/L, LDH 639 U/L, CPK 5062 U/L, aPTT 40.45 sec, haemoglobin 13 g/DL, Platelets 100 × 10^9 ^L^-1^.

On day 7 of admission (5 days after the initiation of antibiotic therapy) his temperature was 38.5°C and treatment was changed with intravenously Ciprofloxacin 500 mg 12 – hourly.

On day 13 of admission the patient became apyrexial, his clinical condition and the results from the blood analysis improved. On day 15 of admission he was discharged on oral ciprofloxacin to continue in the community. No severe complications of intestinal hemorrhage or perforation were observed and, even though S. paratyphi is almost likely to cause chronic carrier state, our patient did not become chronic carrier.

## Conclusion

Our case emphasizes the potent spread of Salmonellae infection in developed countries from infected immigrants. We advocate the need for a high degree of clinical suspicion, an early diagnosis and prompt institution of effective antimicrobial therapy to decrease the mortality, the morbidity and the possibility of outbreak, especially with a multi-resistant organism.

To our knowledge, this is the first report of enteric fever in Greece associated with Salmonella ser. Paratyphi A.

## Abbreviations

Na: Sodium; SGOT: Serum glutamic oxaloacetic transaminase; SGPT: Serum glutamic pyruvic transaminase; LDH: Lactate dehydrogenase; CPK: Creatine Phosphokinase; aPTT: Activated partial thromboplastin time

## Consent

"Written informed consent was obtained from the patient for publication of this case report and accompanying images. A copy of the written consent is available for review by the Editor-in-Chief of this journal."

## Competing interests

The authors declare that they have no competing interests.

## Authors' contributions

CA analyzed and interpreted the patient data and was the writer of the manuscript. AD and TS contributed to acquisition of data. All authors read and approved the final manuscript.
